# A tale of two cities: London and New York City during Covid-19

**DOI:** 10.1371/journal.pone.0305330

**Published:** 2024-09-23

**Authors:** Augustin de Coulon, Marc Scott

**Affiliations:** 1 Dept of Economics, Business School, King’s College London, London, United Kingdom; 2 IZA, Bonn, Germany; 3 Dept of Applied Statistics, Social Science, and Humanities, Steinhardt School, New York University, New York, NY, United States of America; 4 PRIISM Center, New York University, New York, NY, United States of America; Touro University, UNITED STATES

## Abstract

Using publicly available data, this paper investigates the diffusion of COVID-19 across neighborhoods in two major cities, London and New York. We link neighborhood demographics to incidence, and we investigate patterns of change over time in conjunction with changing policy responses to the pandemic. By comparing and contrasting these two cities, we are able to exploit surveillance and policy differences, demonstrating how each contributes information to the other. We conclude that better coordination can be translated into improved health policy.

## Introduction

The first case of COVID-19 was identified in December 2019 [1], in Wuhan, China. The reaction of governments varied greatly, and air travel continued, reasonably normally, until late January, 2020, when for example, British Airways cancelled all flights to and from Wuhan, as cases spread throughout Europe. London, UK, being a hub of international travel, detected its first cases on February 11th. New York City (NYC), despite some travel restrictions, became one of several early hotspots in the USA by March. The focus of this paper is how policy coordination across the two international cities could be improved in order to respond more efficiently to control the spread of the viral outbreak [2].

News outlets have tracked the outbreak and contrasted cities using publicly available data, but typically during single waves [3], without statistical models, and not with systematically linked results as we conduct in this paper. Many studies examined the *effects* of the pandemic from the perspective of race/ethnicity, gender [4, 5] or social class [6, 7] developing crucial support for policies and actions. We consider instead the *spread* of the disease through communities, which would inform policies while the damage occurs [8]. Like [9–11] our analysis includes demographics that characterize neighborhoods and contrasts the relationship between these and incidence over time [12]. Unlike those authors, we simultaneously study two major cities, which allows us to borrow statistical strength and model when the relationships differ [13]. Comparing and contrasting London and NYC over time, we form a dynamic narrative of how the spread could be used in real-time to inform more responsive, preventative and coordinated policies [14, 15].

We track the progression of this disease over the course of two years, aligning a set of publicly available, demographic measures across the cities to build a harmonized data set. We limit our analysis to surveillance data—publicly available data released by the Greater London Authority (GLA) and NYC’s Health Department (DoH). We use a coordinated set of spatio-temporal analyses, eventually pooling results in a manner that allows each city to contribute maximally to our understanding of the disease’s progression through communities. At different times, each city provides more precise information than the other, and we exploit this, borrowing strength across sites to establish the overarching patterns that could benefit coordination between the two cities’ public health authorities [16].

By synthesizing the relationships between demographics associated with multiple neighborhoods and incidence, we build a unique narrative of the progression of the virus through time and place. The observed similarities and differences, as well as our method for combining information, suggests that policies that respond to surveillance across multiple sites would benefit from closer coordination or a global, rather than local perspective. This inter-city collaboration could take the form of cooperative agreements or communications frameworks [17].

### Why study these two global cities?

Coordinated disease surveillance is most meaningful when cities with similar population, economies, and transportation networks are examined. London and New York City have equivalent population size (8.8m for NYC; 9m for London in 2020), they play comparable roles in the world (as cultural hub, tourist destination, and financial center). Both cities share comparable geographic locations, as international gateways to vast areas (Europe and the continental US, respectively). Over time, this generated similar population development and a corresponding labor market associated with the role.

Both cities are demographically similar but not identical. London is 60% white, while NYC is 40%. Their immigrant population is, however, similar in size at 35–40% of the total designated “foreign born”. Historically colonized or formerly enslaved populations make up a significant fraction of the non-white population, with about 25% Black in NYC, and a similar fraction non-white from former colonies (e.g., India, Pakistan, Bangladesh and the Caribbean) in London. Another notable commonality is their mass transport systems, each serving a different population density, with NYC’s being more dense. Both display significant variation in population density, with more suburban areas in outer London boroughs and in Staten Island and Eastern Queens, NYC.

Characteristics such as these likely correlate with differences in exposure and thus incidence rates; the timing of the introduction of variants should also play a role. Our aim is to quantify the relationship between neighborhood demographics and disease progression, with an emphasis on the extent to which they may collectively explain cross-city similarities and differences. We take as premise that geographic variation most likely arises from diverse, if interrelated, sources [18]. Mass testing is crucial for neighborhood surveillance, and the process by which individuals are authorized or required to test differed between cities, particularly in the initial phase of the pandemic. The more general story involves exposure differences linked to work and family responsibilities, healthcare systems, and personal choices reflecting priorities or a sense of responsibility towards one’s community. Our methodological approach reflects two objectives: through surveillance, to learn as much about the course of the disease as possible; through statistical methods and data combination, to understand how cities and their governments can learn from one another dynamically.

Herein, we develop geo-spatially adjusted models for virus incidence as a function of neighborhood characteristics, linking the information across cities, and noting how the relationships—and thus affected populations—change over time. We begin with a comparison of city demographic measures and their interrelationship, finding them roughly similar in perhaps surprising ways. We relate these characteristics to disease incidence over time, building a time-series that establishes which types of communities were affected the most and least, and whether the experiences were aligned. We then organize a set of longitudinal analyses into eight key periods and give a brief overview of overarching policies in each city at the time. These associations allow us to assess more precisely the alignment of disease progression, borrowing statistical strength across studies to establish the pattern. Identifying whether a “current phase” is aligned or not with a “sister city” and the effectiveness of recently adopted policies supports the formulation of a dynamic policy. The sections are organised as follows: data description and methodological overview, findings and synthesis. We conclude with a discussion of limitations in the data and level of analysis, referencing additional data sources to corroborate some of our findings.

## Materials and methods

We provide in an S1 Appendix a full description of the data used, their sources and how they are generated. Here we only list the variables and demographics used. We chose a range of available measures, common to the COVID-19 literature, which were harmonized (made equivalent, or nearly so) across cities, limiting our choices somewhat. While ensuring that key social-determinants were included, we realized that many were correlated with each other, so inclusion of some would be redundant. This applied, for example, to household crowding, which was highly correlated with poverty in London (correlation was .73 with our poverty measure). All covariates are expressed as the percentage with the characteristic for each neighborhood (983 MSOAs in London and 177 ZIP codes in NYC). The demographics included are essential workers (Essential); degrees holders (BAplus); age indicators: residents older than 65 (PercentOlder) and younger residents (PercentYounger); health indicators of: chronic obstructive pulmonary disease (COPD) and obesity (Obese); residents who main spoken language at home is English (OnlyEngAtHome); race/ethnicity: white (PercentWhite), Bangladeshi/Pakistani (in London) and Latinx (in NYC), (coded as PercentHispanic in both cities), Black (PercentBlack); and median income (MedianInc; not expressed as a percentage). Detailed discussion of these covariates and their interrelationships is given in the S1 Appendix. Note that our surveillance data is collected at different geographic unit levels (ZIP contains 50k households, MSOA 9k), a limitation that we address through various analytic techniques through the choice of fixed effects and our regression-based synthesis.

To track the spread of the disease between different types of neighborhoods, we build two types of Poisson regression models for COVID-19 case counts. Notationally, the count for areal unit *i* within larger unit *j* in period *t*, *Y*_*ijt*_, is a function of one of eleven time-constant characteristics of each neighborhood, denoted by *X*_*ij*_. For example MSOA *i* is contained in Local Authority (LA) *j*. The general form of the model for the incidence rate λ_*ijt*_ is:

$$\begin{array}{c} \text{log}\;\lambda_{ijt}=\text{log}\left(n_{ijt}\right)+b_{0}+b_{X}X_{ij}+S\left(t\right)+\delta_{j}+\alpha_{ij} \end{array}$$ (1)

where *S* is a cubic polynomial capturing trend over time, *X*_*ij*_ is the predictor of interest, *δ*_*j*_ is a fixed effect for large regions, and $\alpha_{ij}∼N\left(0,\sigma_{\alpha }^{2}\right)$ are random effects for smaller regions. The observed counts are Poisson: *Y*_*ijt*_ ∼ *Pois*(*n*_*ijt*_λ_*ijt*_). We control for exposure by including the log of the number of residents, which we denote *n*_*ijt*_. (The index *t* allows government agencies to adjust population counts, but in practice, they are fixed for the duration of the study. This number drops when people die, but this effect is minimal).

In the first form of the model, we fit separate regressions for each week of the study and then monitor the adjusted correlation coefficients as a time-series, with 95% confidence bounds based on the s.e. of the estimate. In NYC the fixed effects are restricted to borough, while in London, they represent Local Authority. The *S*(*t*) term is omitted in this pooled cross-sectional time series. This provides a real-time profile of the types of neighborhoods being affected and trends or “spikes” that may be connected to policy changes or events. To the extent that one city’s profile mirrors the other, perhaps lagged or after a similar event, this form of surveillance allows cities to learn from each other, anticipate waves, and reduce disease spread.

In the second form of the model, we pool 10–20 weeks in groups based on eight key periods of the study (roughly based on the four seasons). In this model, we include random effects for ZIP codes and MSOAs. The repeated measures reduce the impact of missing data in London through the random effect and smooth trend, as most intervals contain some periods of high incidence and thus low missingness. With precision gains, we compare seasonal profiles across the cities, providing statistical evidence of alignment. In the NYC models, we include a different set of fixed effects for 11 borough-specific geographic areas built from a larger set constructed by New York Health and Hospitals (NYHH), while we continue to add fixed effects for the 32 Local Authorities in London. The choice in NYC is an attempt to have a sufficient number of smaller areal units inside of the larger one, while still capturing broad regional differences, given ZIP code constraints, so demographic differences between smaller areas are to be interpreted as *relative*. As well, London data are provided at a much more local level, and this is an attempt to keep the models as similar as possible in terms of the ratio of fixed (larger region) versus random (smaller region) effects.

In both models, the parameter of interest may be interpreted as a correlation adjusted for neighborhood differences. For example, Manhattan residents on average have higher income than those in Staten Island, and they may have had lower incidence rates in the first year of the pandemic. Controlling for these differences, how does income *within borough* relate to disease incidence?

### The Weighted Least Squares approach

In order to understand the relationship between measures, over time, and across cities, as well as limit any effects of multiple comparisons, we combine the findings, borrowing statistical strength often from London, but sometimes from NYC. Combining the findings into a single summary statistic for each of eight periods informs how more coordinated health policies could improve decision making.

This is achieved by correlating the coefficient estimates of neighborhood characteristics for two cities into eight unique figures using a Weighted Least Squares (WLS) model, quantifying the direction and extent to which our 11 coefficients are aligned. In the WLS, we use London estimates as the outcome and the corresponding NYC estimate as the value of the predictor. We summarize and synthesize the period-specific relationship between the adjusted effects for our demographics using a Weighted Least Squares (WLS) approach, with London effects as the outcome and the corresponding NYC effect as the predictor. This approach can be summarised following regression:

$$\begin{array}{c} y_{it}^{L}=\alpha_{t}+\gamma_{t}x_{it}^{N}+\varepsilon_{it} \end{array}$$ (2)

for demographic indexed by *i* and sub-period indexed by *t*, and $\varepsilon_{it}∼N\left(0,1/{\hat{\sigma }}_{it}^{N}\right)$

To be clear, *fixing the sub-period t*, we set $y_{i}={\hat{\beta }}_{i}^{L}$ and $x_{i}={\hat{\beta }}_{i}^{N}$ be the 11 point estimates (from Figs 2 and 3, London and NYC, respectively). NYC weights were used because NYC precision is much lower. We run separate regressions for each sub-period, resulting in eight regression lines, which will be presented in Fig 4, in which estimated coefficients taken from Figs 2 and 3 described next are the data points. Symbol colors are coded to reflect significance in NYC only (blue), in London only (red), in both (black) and in neither (empty). So, for example, in Summer 2020 we can observe that areas with larger share of degrees holders (BA+) in both cities were less likely to be affected (hence black *dot*: Both), areas with greater shares of Latinx (NYC) and Bangladeshi/Pakistani (London) were displaying greater COVID-19 positive cases, but only significantly so in NYC (hence blue *dot*: NYC only). Overall, the relationships between the estimated coefficients correlates positively. Standardized, the correlation is greater than 0.7 for all significant periods, suggesting the potential benefit of coordination between the two cities’ health authorities.

## Results

### Disease incidence over time

**Fig 1:**
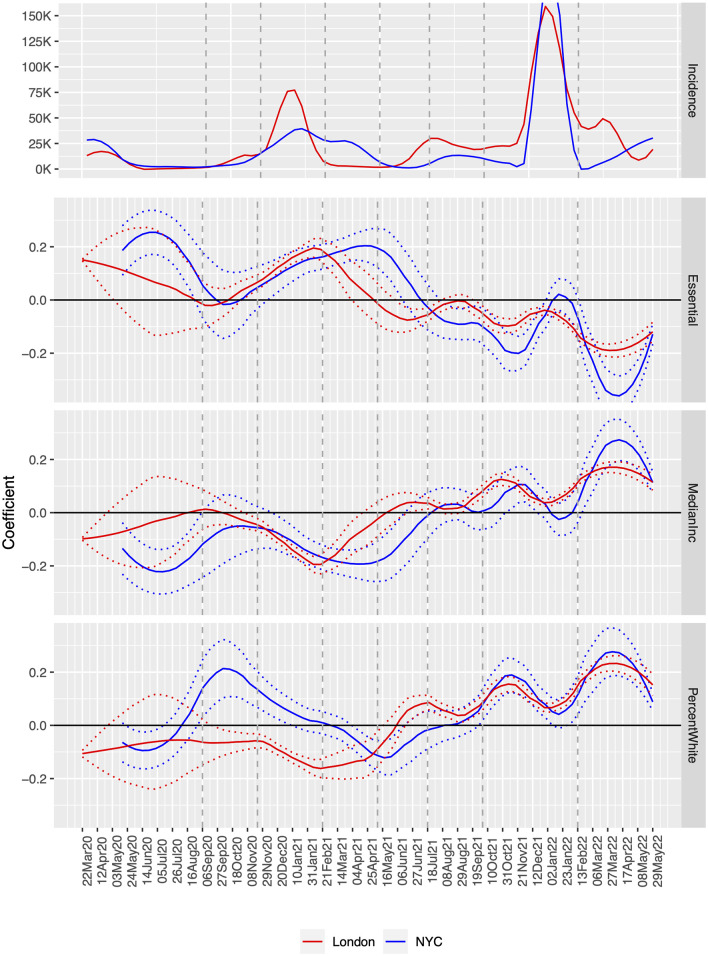
Adjusted correlates over time. Note: The lines are based on coefficients from estimating Poisson models (Eq 1) using respectively 983 MSOAs and 177 ZIP codes and the 3 characteristics: Essential workers (Essential), Median Income (MedianInc) and Percent White (PercentWhite). The curves are derived using LOESS, a non parametric smoothing function.

We investigate incidence over time by generating the time-series of adjusted effects (Eq 1) for all 11 predictors and plotting the resulting series, three of which are displayed in Fig 1. At the top of the plot we show disease incidence (for reference, with dashed lines delineating key periods, found in the ‘Eight Key Intervals’ discussion), followed by effects for the percentage of essential workers in the neighborhood, the (log) median income of the neighborhood, and the (log) percent white. This choice was based on the fact that the other correlates reveal similar or mirror-image trends, which we attribute to inherent correlations between demographics, even when the domains are different. Another reason to highlight these three is because they are plausibly less threatened by the ecological fallacy due to their high variability across areal units. To illustrate this variability, we compare 90th and 10th percentile ratios of these demographics. For median income, this ratio is 2.14 for London and 3.22 in NYC. For percent white, it is 2.55 in London and 7.20 in NYC. For percent essential worker, this ratio is 1.76 for London and 2.30 in NYC.

The five waves (initial, Alpha, Delta, Omicron BA.1/2 and Omicron BA.4/5) are peaks in the top panel. London incidence was under-reported in the first phase due to testing policy. For this portion of the incidence plot we made a minor correction to align the *aggregate* counts using the ratio of hospitalization to cases in NYC to calibrate London data, which for the most part reflected hospitalizations. London waves tend to precede those of NYC, and the Alpha and Delta waves are examples in which NYC incidence was lower, but sometimes lasting longer. *This may reflect policies that reacted to global surveillance*; we contend that such information sharing could be used more effectively with analyses such as ours.

The adjusted correlations identify vulnerable or more affected populations, offering more precise targeting of interventions, particularly because the virus moves through populations in both cities in a similar manner. For example, essential worker neighborhoods account for more cases in the first year and a quarter of the pandemic, which is consistent with exposure due to their occupation. The vaccine introduction at the start of 2021 appears beneficial for these workers, as the coefficient wanes even into the Omicron wave. Income is a nearly mirror image of essential workers, both between and within cities, with more economically advantaged communities at lower relative risk for the first part of the pandemic, as they likely worked from home or left the city [19]. At the end of the Summer in 2020, the coupling between the cities changed with respect to income, but it returns a few months later as both cities roll out vaccination efforts.

The differences between cities is also revealing: white neighborhoods in both cities have “spikes” that belie the overall trend associated with economic roles and resources. During the Summer of 2021, in London, a spike in cases in white neighborhoods coincides with documented travel associated with a major football event [20]. A huge incidence increase in some of NYC’s predominantly white neighborhoods in Fall 2020 coincides with political and religious backlash against masking and other restrictions at the time of a US national election [21, 22]. The Delta wave hit London before NYC, so that by the Summer of 2021 (and after the football event) London’s neighborhoods with larger white populations were more adversely affected. As the Omicron waves hit, both cities’ largely white neighborhoods have correspondingly large increases. While the Omicron variant was purported to affect everyone, relatively speaking, white wealthier neighborhoods were areas of higher incidence. Paradoxically, these were often neighborhoods with higher vaccination rates, perhaps inducing a false sense of security given the ability for this variant to bypass the immune system. We cannot rule out the possibility that some populations are reporting test results more than others, due to the availability (and in the case of NYC cost) of at home tests and a reduction in public testing sites. The context of testing in the early phase of Omicron in NYC is discussed in [23], while changing conditions in London are discussed in [24].

We contend that these cities can learn from each other as the disease progresses. London typically “leads” NYC by 2–4 weeks, particularly for the essential worker and median income effects. One can often predict which neighborhoods, and in some sense, *who* will be affected next in NYC by examining London today.

### The eight key intervals

A limitation of time-series models is their lack of precision and pooling—they are cross-sectional analyses calculated weekly, then smoothed to identify strong trends and deviations. Periods of larger and smaller incidence appear as waves, as do correlates of incidence, since different populations are more or less affected in each wave. We organize this next analysis by pooling larger time intervals, each approximately one season, to both gain statistical power and minimize the effects of intermittent changes, establishing more definitively the pattern of community spread.

**Fig 2:**
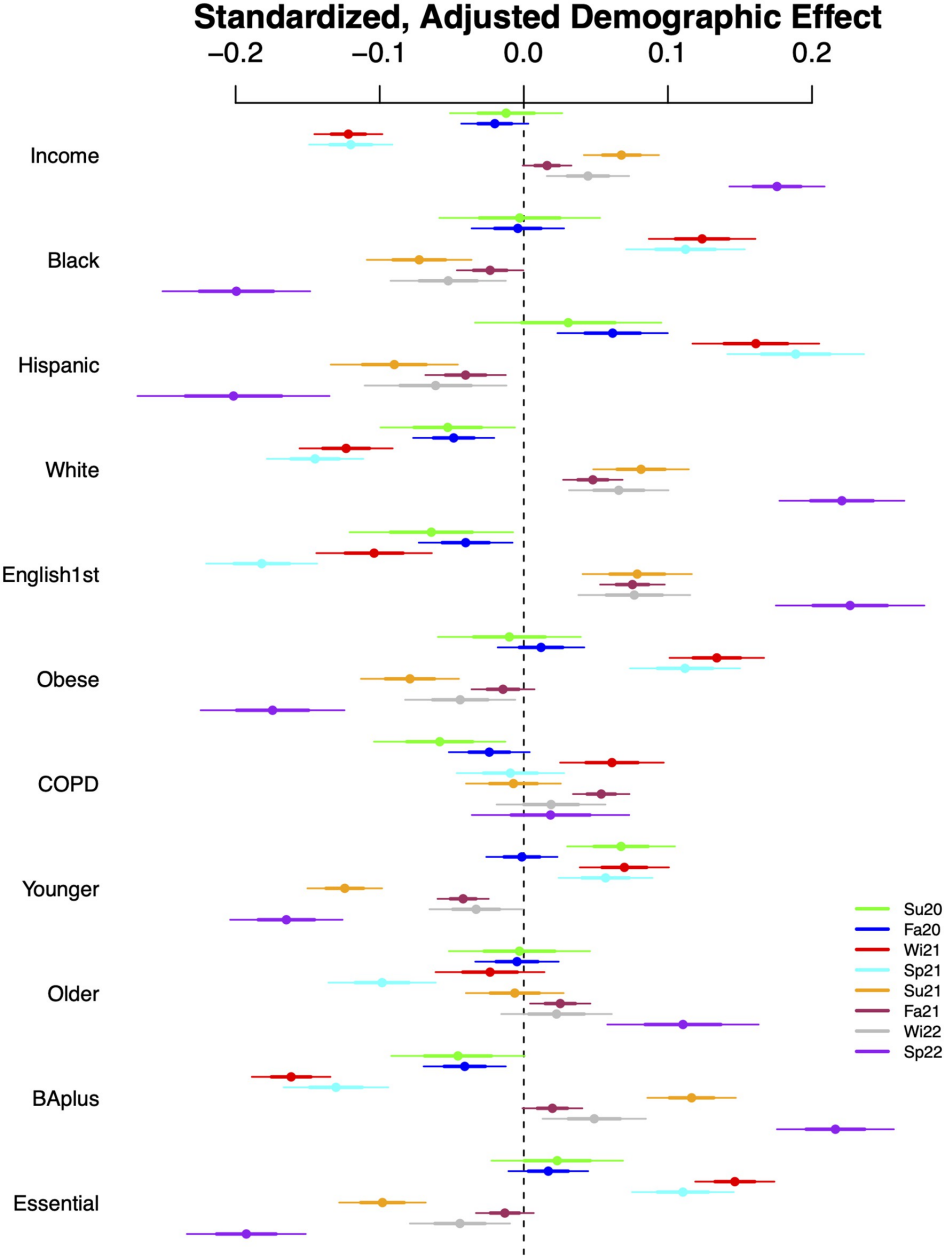
Adjusted regression coefficients: London. Note: Here Eq (1) is used by pooling the week within each period. The S1 Appendix explains and discusses the choice of the eight time periods: Su20 is for Summer 2020, Fa20 is for Fall 2020, Wi21 is for Winter 2021, Sp21 is for Spring 2021, Su21 is for Summer 2021, Fa21 is for Fall 2021, Wi22 is for Winter 2022 and Sp22 is for Spring 2022.

**Fig 3:**
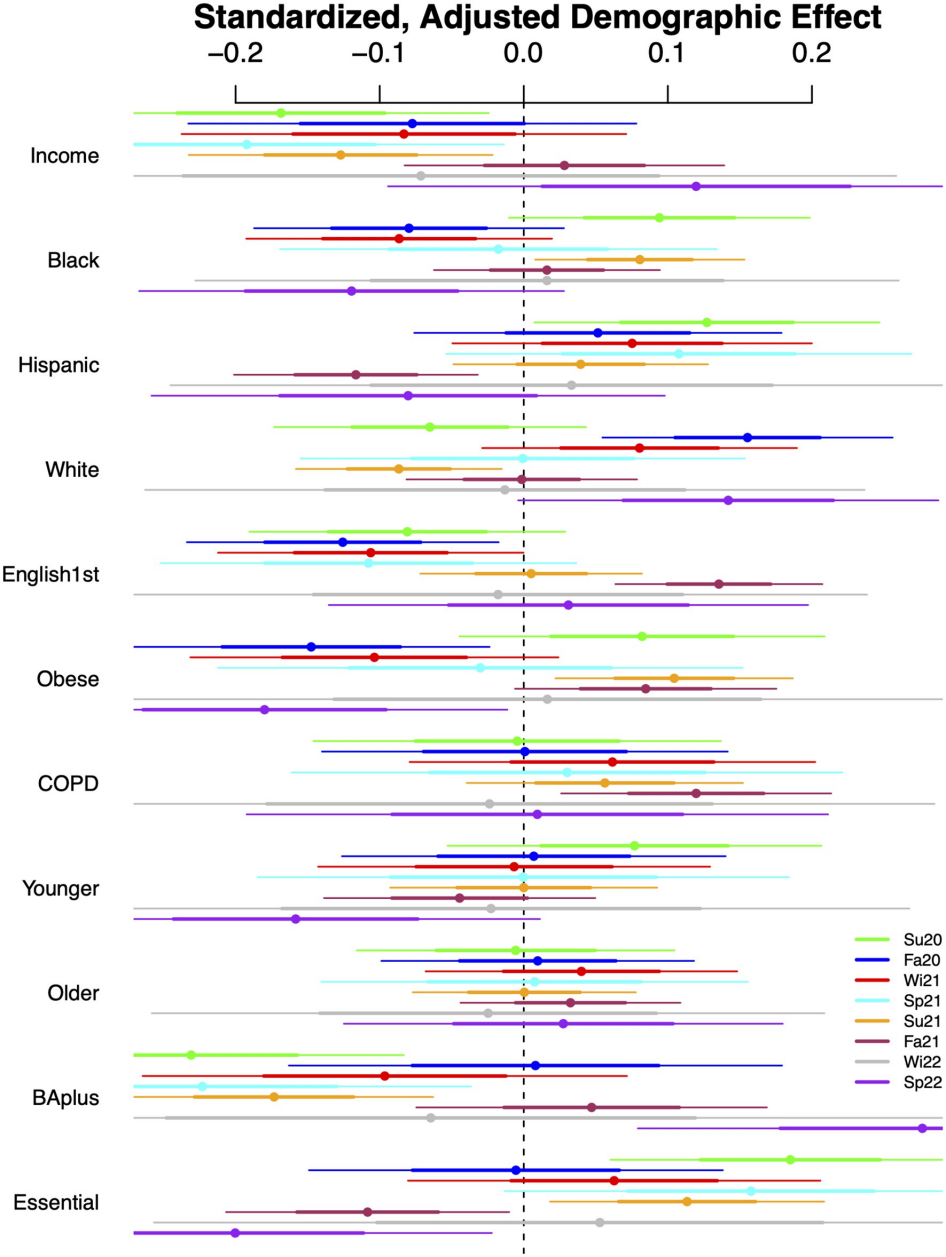
Adjusted regression coefficients: NYC. Note: Here Eq 1 is used by pooling the week within each period. The S1 Appendix explains and discusses the choice of the eight time periods: Su20 is for Summer 2020, Fa20 is for Fall 2020, Wi21 is for Winter 2021, Sp21 is for Spring 2021, Su21 is for Summer 2021, Fa21 is for Fall 2021, Wi22 is for Winter 2022 and Sp22 is for Spring 2022.

In Figs 2 and 3, we report the adjusted regression coefficients for London and NYC, respectively, in eight key periods (details given in the S1 Appendix). Each row corresponds to a different demographic, and within the row, the ordering, top to bottom, is from Summer 2020 to Spring 2022, with colors used to enable comparisons of the same period across measures (legend just above bottom right). The center point is the estimate, the inner and outer intervals are one and two standard error bounds, respectively.

For London, we have the striking finding that income emerges as an important protective effect in the third period, substantially after the initial community spread, followed by a stronger positive relationship in most of the second year of the pandemic. This stands in stark contrast to the neighborhoods with relatively more Black or Bangladeshi/Pakistani populations; they are nearly mirror images. Essential worker neighborhoods are positively correlated with these two race/ethnicity groups, and that is reflected in the corresponding coefficient plot. In London, obesity follows this same pattern, perhaps more due to correlation with demographics than to health reasons, per se. COPD is less definitive, but its variation at the MSOA level is low. Interestingly, the effect of age does emerge, but it is to be understood as reflecting (mirroring) the correlation with essential worker neighborhoods, which tend to be younger in London. Older neighborhoods may have avoided high incidence in Winter 2021, which was the start of vaccination, with that population queued to receive it first. The more educated follow the pattern of the white and higher income neighborhoods quite closely. In sum, London neighborhoods reveal very specific trends, but these duplicate or mirror (reflexively) each other for the most part.

For NYC, we witness the decreased precision of NYC estimates, even pooled across a season. While only one-quarter of the effects are significant at traditional levels, we will soon borrow strength from the precision inherent in London’s estimates to build a comprehensive narrative and comparison. We note that higher income neighborhoods were associated with lower relative incidence early in the pandemic. Latinx neighborhoods are qualitatively similar to those of essential workers, but less precisely so. In those neighborhoods, initially higher positive association was followed by negative, indicating a shift away from those populations. Somewhat striking is the variation over time in white neighborhoods, with a spike in cases during the Fall 2020 period. While there is some variation over time in our two health measures, obesity and COPD, the low prevalence of those measures and their correspondingly lower variability across neighborhoods make us reluctant to draw strong conclusions. The lack of local geographic diversity in age in NYC likely accounts for non-significant effects centered near zero. Neighborhoods with larger college educated populations, for which there is great variability in NYC, were initially lower incidence; we posit this is due to working from home. This shifted to higher relative incidence, especially in the last Omicron wave; we posit this is due to full re-opening and return to office.

Given the geographic constraints, particularly the areal unit size, adjusted standard errors for the effects in NYC are more often non-significant. To be more precise, across all measures and intervals, 73% of London effects are significant, while only 26% of NYC effects are. This obscures important variation connected to differences between the two cities. For example, in both cities, essential worker and college education effects are significant in at least half of the key intervals, whereas age is never significant in NYC, but percent younger is significant for London in 7 of the 8 intervals. Exploring this a bit further, we find that NYC boroughs (larger geospatial units) are quite different demographically with respect to age, while this is not true in London, and thus our NYC models already control for age somewhat through fixed effects.

### Multiple comparisons

While we limited the number of demographic features to 11, we still have some concerns about issues of multiple comparisons. Each of the eight intervals is of interest in its own right, and non-significant effects during a period may be considered as evidence of complete community spread (e.g., Omicron wave), but to be conservative, we applied an adjustment for false discovery rate [25] to each city and interval. Applying an alpha level of 0.05 to the family-wise adjusted p-values had minimal effect for London, with effectively no change in significance other than in the Summer 2020, for which only one effect remains significant. This interval contained significant missing data (and thus decreased precision) in that city. Our findings change in NYC, for which the number of significant effects halves in Summer 2020; it had little effect in Summer and Fall 2021. The remaining periods, adjusted, almost never yield a significant correlate in NYC.

**Fig 4:**
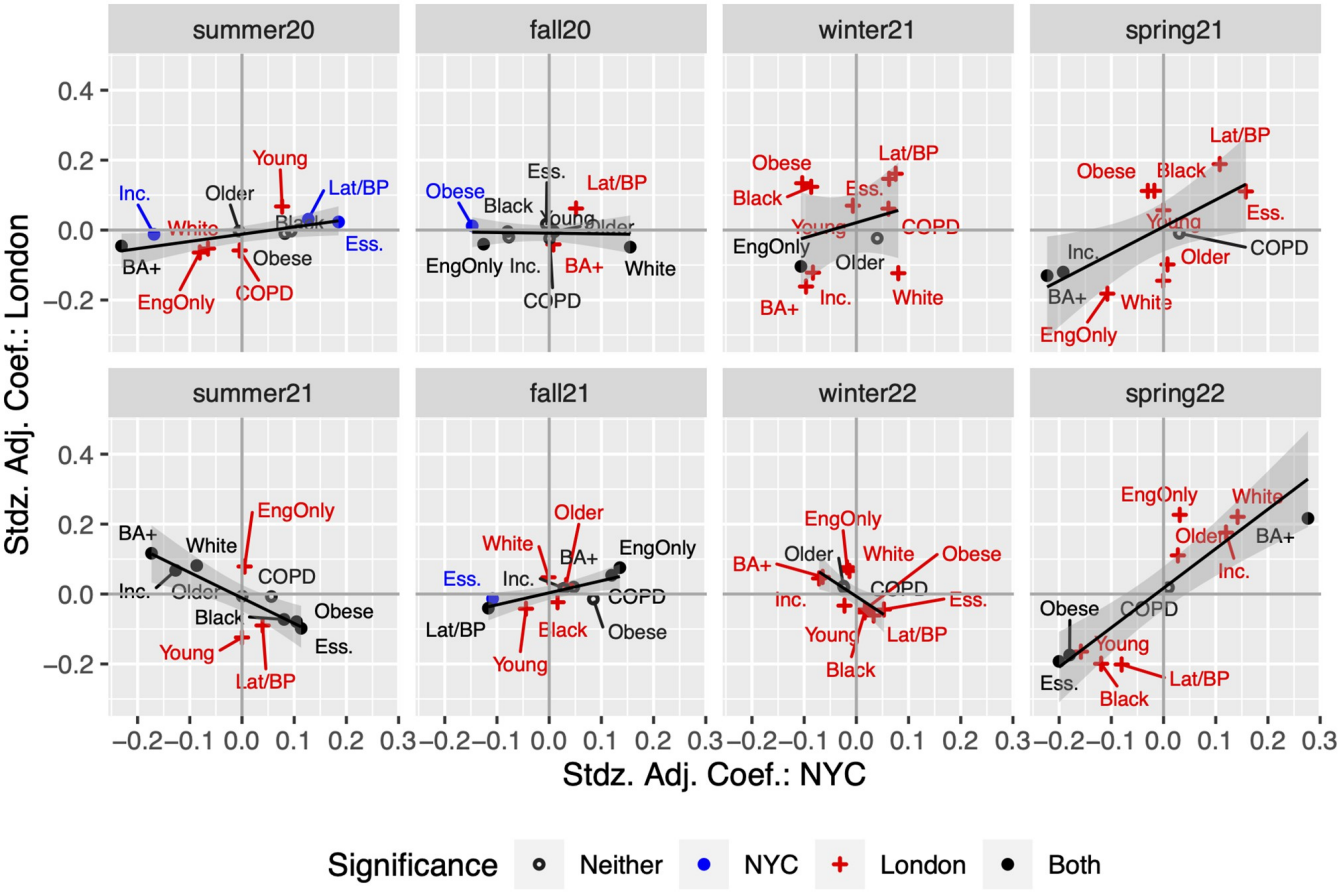
Relationship between regression coefficients: NYC and London. Note: Each dot is a coefficients taken from Figs 2 and 3. The regression lines are from Eq (2) where London effects are the outcome and NYC the independent variable. Dots in black (filled) means they are both significant for London and NYC, in blue significant for NYC only, in red significant for London only and black (hollow) neither significant for London nor NYC.

Across the eight periods, we find that for Summer 2020, Spring 2021, Fall 2021, and Spring 2022, the standardized slope of the regression line is positive and significant, comparable to a correlation, with values 0.71, 0.74, 0.84, and 0.94, respectively. The relationship reverses to negative and significant in Summer 2021 (-0.82) and Winter 2022 (-0.80). No significant relationship is identified in periods Fall 2020 and Winter 2021. A positive relationship implies that similar populations have greater incidence relative to their neighbors in both cities; the virus is spreading across similar communities/demographics. The community/demographics spread changes over time, but it changes in the same way across cities when the sign is positive. A significant negative relationship likely occurs when a major policy or community behavior differs. We summarize the relationships in Fig 4; a detailed discussion follows.

#### Summer 2020

This is the first wave, and the neighborhoods with the highest relative incidence tended to be more Latinx in NYC, corresponding to Bangladeshi and Pakistani in London (the latter’s coefficient was not significant). There are also more essential workers and younger people in these neighborhoods. Conversely, neighborhoods with more college educated, white, higher income, and English at home had lower incidence *in both cities*. We posit that these relationships were smaller in magnitude in London due to measurement error induced by selection. Another factor may be data censoring in London, but this should have an affect on precision, not magnitude, and both are impacted.

#### Fall 2020

The linear relationship between estimated effects for NYC and London is not significant, and as in the prior period, NYC effects tend to be much larger. Concerns over statistical power limit what we can conclude from this phase, but most notable is the significant positive effect for neighborhoods that are proportionally more white. This is the period leading up to the November 2020 election in the US, with substantial anti-mask sentiment during a period of ‘re-opening’. It is a period of reasonably low incidence, so strong effects suggest certain populations’ behavioral differences.

#### Winter 2021

In this period of increased incidence (Alpha variant), we have patterns in London that resemble the first wave, in which essential worker, Bangladeshi and Pakistani, and younger neighborhoods have higher incidence (these are often the same neighborhoods). Similarly, more college educated, white and wealthier neighborhoods experienced relatively lower incidence. The relative nature of this finding must be emphasized particularly because it is in the context of a long and sustained wave. The wave began with a pre-Christmas lockdown in London that backfired as cases soared, while NYC experiences a weaker but more sustained wave.

#### Spring 2021

This period has many of the same features as the first wave in terms of affected populations, but the relationship between London and NYC is stronger and significant, allowing us to borrow strength across cities, and posit that spread in NYC is similar to that of London, with few significant NYC coefficients. Thus, the relatively more affected populations in both cities are in essential worker communities, which are Latinx and Bangladeshi/Pakistani as well. Communities with larger fractions of college graduates and higher incomes are less affected. It is a period with fairly similar policies of a full reopening in each, and reasonably similar vaccination roll-outs.

#### Summer 2021

In this phase, there is a reversal of relationships, meaning London and NYC have opposite signs for key demographics, and the relationship is significant. Communities that are more educated, white and wealthy are at higher risk, relatively speaking, in London, while these same communities remain lower incidence in NYC. The full force of the Delta wave hit London in this period, and these communities, vaccinated or not, under few restrictions, suffered substantial community spread. The Delta wave hit NYC later in the summer, but the communities affected were more non-white and essential workers. NYC data at this point revealed that incidence was much higher among the unvaccinated [26].

#### Fall 2021

This phase is really the late summer/early fall, and while NYC now has the stronger relationships, the effects are often significant in both cities. There is a realignment in which communities are more at risk, shifting to those with English at home and away from the Latinx/Bangladeshi/Pakistani and essential worker neighborhoods. The London/NYC correspondence is strong, as judged by the significant and larger correlation (0.84), but a lack of variability in London suggests a more general community spread in this pre-holiday travel period, with limited public restrictions [27, 28].

#### Winter 2022

This is the Omicron wave in both cities. It is characterized as a significant *negative* relationship across cities, but importantly, the effects are small across nearly all demographics. This suggests widespread incidence regardless of *who* is living *where*. NYC effects are all non-significant; while the dynamic movement of the virus between communities was more complex during this period, the speed and magnitude of the spread is not precisely captured in NYC. More precise measurement in London suggests that the populations more at risk have shifted once more, toward the more educated, white and wealthy communities, with English spoken at home. That tendency is not apparent in NYC—yet.

#### Spring 2022

The Omicron subvariant (BA.4 and BA.5) waves hit both cities, with NYC lagging behind London by about 3–4 weeks. There is sufficient evidence, however, that both cities are largely aligned in terms of which populations are relatively more at risk. These are the more educated, white, wealthier communities in both cities, with NYC having “caught up” to London. We must underscore that at this phase of the pandemic, testing has become somewhat more voluntary, and vaccination passes have been all but eliminated in London (indoor performances still required masks in NYC). This has the potential to introduce stronger selection bias in who gets tested or reports their home test, given changes in policy and limited enforcement.

## Discussion

Our findings suggest that we are tracking how the virus itself spreads through communities and under what circumstances. We built a neighborhood surveillance tool using publicly available data, adjusting demographic correlates so that they capture *relative* differences in incidence across adjacent neighborhoods. Our model specification, mass testing, and demographic variability across neighborhoods decreases the risks inherent in ecological inference, but the interpretation as a neighborhood effect still holds. The challenge of making policy informed by heterogeneous units remains. This is where the idea of borrowing strength within and between studies emerges. If a similar demographic is more prevalent in neighborhoods with higher risk, we can cautiously infer from the neighborhood level to the group. In fact, since many of our demographics are split across multiple indicators (each race group, e.g.), we learn whether relative differences are important.

In terms of policy levers, we argue that information campaigns could be customized to different populations, for example in terms of language or cultural practices. Thus, while we could not, and would not try to change the demographics of a neighborhood, we could identify subpopulations that appeared particularly vulnerable, and have intervention campaigns targeting them. Demographics, combined with knowledge of communities and their public health histories, can inform us about potential mechanisms and how these can be used to develop policy responses.

Focusing on key periods of the pandemic, we demonstrate how public health authorities in both cities could have anticipated the evolution of the disease sooner and with more precision and how this connects to policy. Consider the second wave in the Winter 2020 and early months of 2021. This was a period with only a handful of vaccinations, very high mortality rate, and very tightly regulated lockdowns during cold winter months, which for many will probably remain as the most mentally straining part of the pandemic. Our comparative plot shows that public health initiatives targeting essential workers (both cities) and Latinx (NYC)/Bangladeshi and Pakistani (London) would have helped raise awareness and potentially reduce transmission rates in those sub-populations. When the Delta wave hit London in early Summer of 2021, NYC had a unique opportunity to limit the scope of that wave, and *it is entirely possible that it did*. In Summer 2021 leading into Fall, the incidence rates shown in the top panel of Fig 1 are attenuated for NYC. Vax-pass and policies were extant at that time, and start of public school was delayed and substantial school-based testing resulting in short closures were common. The negative slope in Fig 4, Summer 2021 panel, indicates that different populations had become at risk, so that behaviors or policies could have been further adapted to accommodate this information. Finally, during the eighth period (Spring 2022 panel, Fig 4), the large effect sizes for more educated and white communities suggest a behavioral public health campaign. Residents of these communities could be reminded of their prior successful behaviors, appealing to the longer narrative of the pandemic. In this period, essential workers seem to be managing their risk well, and this applies to both London and New York.

We also learn indirectly about ill-informed policies and their effects. The timing of the Christmas lockdown in London (Winter 2021 period beginning late 2020) arguably increased the extent and duration of that wave [29]. Lax enforcement of laws intended to stop the spread in NYC prior to the Fall 2020 elections coincide with an anomalous “spike” that is concentrated in more white and politically conservative neighborhoods. It could also be argued that by reopening London pubs and many stadiums before the young were vaccinated in Spring 2021 (the pub/football effect) implicitly implemented a policy of unleashing the virus on the young (and predominantly male) in the UK [30, 31].

Other studies confirm or supplement many of our findings, particularly when variability or ecological inference limited them. Age is an example in which low variability across neighborhoods prevented us from identifying an effect that other data have confirmed [32]. Namely, that for many periods during the pandemic, older individuals had lower incidence rates, presumably because they became aware of their increased risk of severe disease response. By the Delta and Omicron waves, younger individuals were more affected; at this point, they were keenly aware of their decreased risk of serious infection. Data aggregated by age group indicate this was true in both cities, and particularly in the most recent waves, suggesting that accumulated knowledge translated into preventative practices and changes in transmission rates [33]. Positive vaccination effects indirectly suggested by our analysis were short lived, but this masks the overwhelming direct evidence that vaccination has a protective effect against infection and more so against severe infection and the need for hospitalization, especially in older populations. As noted, segregation of neighborhoods along race lines is severe in both cities, and when our neighborhood measure of race/ethnicity revealed a spike, it could be confirmed from public data aggregated by race. Other studies relying on alternative data collection techniques are consistent with aggregate analyses, e.g., [34] uses mobility networks and confirms the essential worker, race, and income disproportionalities reported elsewhere.

This research demonstrates the potential for differentiated and potentially more successful public health campaigns targeting specific sub-populations at city levels. The advantages of this approach include its relatively easy implementation relying on aggregate data at low (MSOA and ZIP) geographical levels. This would be of course less costly than community surveillance performed on individual longitudinal samples such as performed in the Coronavirus Infection Survey [35, 36] in London. Indeed the COVID-19 survey’s reduced sample size (N=51,113) [37] precludes public health targeting of neighborhoods. Similarly another ecological analysis [34], surveilles using cell phone data, but data restrictions limit the wide applicability and transparency that our approach enjoys. We rely on public health systems implementing mass testing, but the anonymization to protect individuals does not preclude us from building evidence to inform policy.

Another advantage of our approach is that conducting surveillance in two cities with similar characteristics (in population size, segregation, industrial specialization, etc.) provides greater confidence in the validity of the results than those observed for a single city and/or conducted independently. We find that differences in data collection meant that NYC, even with its larger geographic units and tendency to lag London, could inform London estimates. While we partitioned the analysis into distinct periods, a rolling window of sufficient size could provide finer grained surveillance with potential for customized public health messages with only a small lag to the quasi-instantaneous spread of the disease. We note that our approach was less useful in the first and particularly the fourth periods (Summer 2020 and Spring 2021), for which the latter’s very small case counts prevent robust and precise assessments of risk to specific sub-groups. However, in periods of very low case counts, the public health risk is low, moderating this concern.

## Conclusion

In sum, our comparative surveillance approach to mapping the disease progression through different populations yields insight into constraints linked to social class and the labor market, human behavior (adaptation and reversion), and perception of risk. The choice of London and New York City is far from arbitrary; rather it can be understood in the broader context of coordination between regions facing similar threats, as made evident in [13, 15]. While there are limitations to what we might learn from a surveillance approach, we contend, as does [12], that it is often essential and expedient to do so. Moreover, many of our stylized facts and interpretations of the findings were validated or informed by other sources and literature. Clearly, this pandemic has taught us that multiple, interrelated factors are connected to substantial differences in risk and outcomes. What seems lacking in the discourse is that we can learn a lot from each other. Each city conducts micro-experiments, intentionally and unintentionally, through its policies and practices, and these are rich with information that could be used to save lives and reduce the burden on healthcare systems.

## Supporting information

S1 Appendix (ZIP)
