# Supplementary material for: A tale of two cities: London and New York City during Covid-19
Source: PLoS One. 2024 Sep 23;19(9):e0305330. doi: 10.1371/journal.pone.0305330 (PMC11419385; doi:10.1371/journal.pone.0305330)
Supplement: S1 Appendix — (ZIP) [file pone.0305330.s001.zip › S1_Appendix.pdf]

S1 Supplementary Information
A Tale of Two Cities: London and New York City during Covid-19

Augustin de Coulon[a,b] * and Marc A. Scott[c,d]

[a]Economics, King's College London, UK
[b]*IZA Bonn, Germany*
[c]Applied Statistics, Social Science, and Humanities, New York University, USA
[d]*PRIISM, New York University*

03/09/2024

*Corresponding author: augustin.decoulon@kcl.ac.uk

Appendices

This Appendix includes a detailed presentation and discussion of the covariates used in the analysis in Section A. We document each, with London presented first followed by NYC. Most references to variable moments (mean, standard-deviation) as well their correlation with other variables are made in the main text, but we present the full set of correlation matrices, aggregated and conditional on region, in Appendix B in Figure S1 and S2. We also introduce tables of descriptive statistics for the two cities in Table S1 London and Table S2 NYC.
We then present the key periods used in our investigations in Appendix C. We emphasize the events in each period that bear direct relevance to the story of the progression of the disease.

A Covariate description

Essential/Critical workers are defined as 'at risk' workers in the UK data. The definition is survey based (The Annual Population Survey (APS) combined with O*NET), where a worker is classified as 'at risk' if their occupation (4 digit SOC) scores high (72 and above) on a 0-100 scale measuring proximity with others (0 is 'no contact' and 100 is 'touching'). We report the proportion at risk. The data is publicly available from the ONS (ONS, 2020). In NYC, such a detailed scale is not available but we have the industries of workers in each geographic area from the ACS (2017). We defined essential workers to include: Retail; Wholesale; Transportation and Warehousing; Entertainment, Accommodation and Food Services; Employment, Health Care and Social Assistance; Employment, Accommodation and Food Services; Production, Transportation, and Material Moving. Jobs in these industries involve either public facing work or 'factory' work that involves a lot of exposure to fellow workers. This list matches closely occupations with high risk of COVID-19 hospitalization and deaths (Chen et al., 2021). We excluded the general service industry, as it is too broadly defined (including many administrative jobs that moved to remote work). Robustness checks did not reveal striking differences with this removal.

Age is separated into two measures, percent younger and percent older, where younger is age 15 (UK) or 17 (US) and under and older is 65 and older in both countries. Age 17 is close to the initial vaccine eligibility cutoff and mortality risk is much higher for those 65 and older; once individuals become aware of their personal risk, presumably they will take appropriate precautions. In making our choices, we were somewhat limited by available age cutoffs from US Census and ONS data, thus the different cutoffs. Note as well that it is possible, but not likely, for a neighborhood to be disproportionately younger and older at the same time (young families living in close proximity with elder housing).

Race/ethnicity

In the UK, MSOA level demographic information was amassed from the 2011 Census. The Black population in London is more broadly defined; we use the percent identifying as Black, African, Caribbean, Black British (as per Census definition). In NYC, anyone with African heritage is identified as Black. Both of these populations have historically suffered from residential segregation that included disproportionate exposure to environmental hazards such as pollution (see Nafilyan et al., 2022; Tessum et al., 2021). Hispanic or Latino may be of any race, and this designation is collected as well. While ethnically very different, the Bangladeshi and Pakistani populations in London are often filling similar roles in the labor market as the Latino population does NYC. For example, they are disproportional represented in the Essential Worker population (The Health

Foundation, 2020).

Income

We initial considered two measures of economic means, percent of the population below the poverty line and median income. We determined that the measures were more comparable across cities using income, as definitions of populations in poverty vary by city, and the welfare state differs somewhat between them.[1] We use the logged version of this measure, as changes in it approximate percentage relative difference, and its distribution is reasonably symmetric in both cities.

Health Risk

At the outset of the pandemic, a concern was that individuals with respiratory problems (e.g., asthma, chronic obstructive pulmonary disease (COPD)) were more likely to have a severe case of the disease. They were thus more likely to have symptoms and get tested, and during the initial phases, selection – who got tested – mattered more. However, health concerns could function in the opposite manner: once recognized as being more at risk, such individuals might change their behavior to avoid infection. In this study, we examine two co-morbitities, obesity and COPD (recorded as Asthma in the UK), taken from NHS Digital (Quality of Care Framework, QOF, 2019) and ACS (2017), as a fraction of the neighborhood population.

Education We operationalize education as percent of the population having a four-year (Bachelor's) degree or higher. This serves as a marker of social class, earning potential, allowance to work from home, and possibly trust in government/scientific authority (see Huang, Brink, & Groot, 2011; Smith et al., 1998). Note that for NYC, the measurement is the percent age 25 or older with a Bachelor's or higher, so the mean, being conditional, is higher.

English Language

In the UK, any household in which no one speaks English as the main language is divided by all households in the MSOA to form the proportion (Census, 2011). To improve comparability across the cities, we take the complement as a percentage, so the London measure reflects the proportion of London households in which at least one member speaks English. In the US this rate is the proportion in which English is the *only* language spoken at home (ACS, 2017).

Other Measures

We considered using a few other measures, but most were highly correlated with others already chosen or were not as clearly connected to exposure. A good example is commute time, which could be long or short for a variety of reasons linked to social class, and the transportation manner itself (e.g., train) may or may not be a primary exposure site depending on masking restrictions/enforcement.

B Correlation matrices and Summary Statistics

Below, we provide correlations between the 11 neighborhood-level demographics, both aggregated and conditional on five borough or geographic regions within each city in Figures 1 and 2. Key correlations taken from these tables are referred to in the main text. These are followed by their summary statistics in Tables 1 and 2. Note that logged values of percent White, Hispanic and Black, as well as median income were used in the study, so they are reported in that form here.

[1]While percent living in poverty measures and (log) median income are correlated at 0.80-0.90, depending on the city, we prefer to use median income for cross-national comparisons and for its distributional properties.

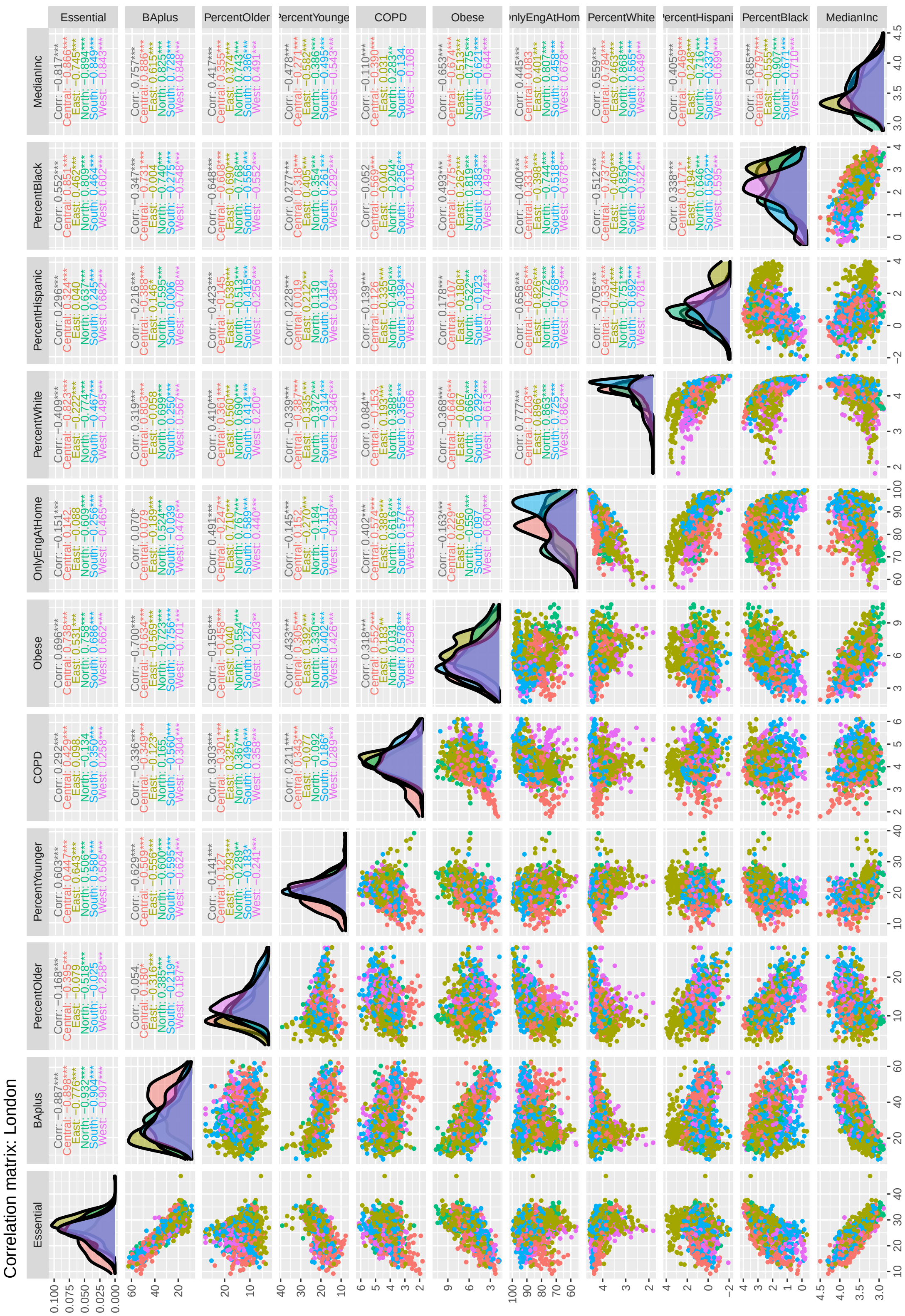

Figure S1: Correlations between key demographics: London

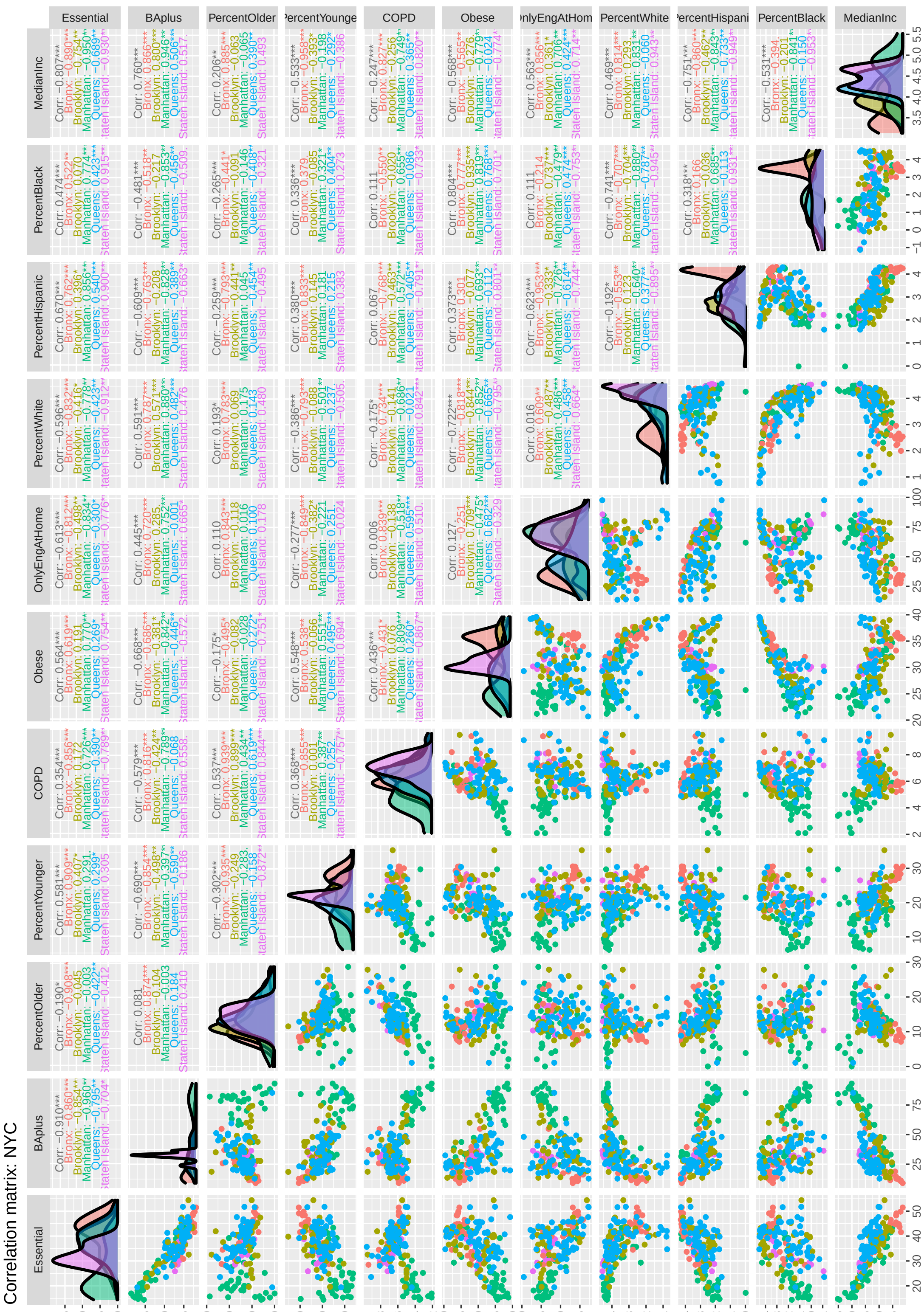

Figure S2: Correlations between key demographics: NYC

Table S1: Summary Statistics: London

Variable	N	Mean	Std. Dev.	Min	Pctl. 25	Pctl. 75	Max
Essential	983	25.651	5.283	9.218	22.471	29.411	46.95
BAplus	983	30.317	11.826	8.19	21.033	38.389	62.617
PercentOlder	983	12.444	4.547	2.497	9.109	15.096	27.659
PercentYounger	983	20.579	3.844	7.721	18.332	22.98	39.222
COPD	983	4.185	0.663	1.8	3.785	4.6	6.12
Obese	983	5.734	1.627	1.73	4.61	6.74	10.69
OnlyEngAtHome	983	84.379	8.948	56.283	77.953	91.484	99.662
PercentWhite	983	4.037	0.402	1.814	3.837	4.334	4.566
PercentHispanic (log)	983	0.988	1.119	-2.084	0.194	1.651	3.979
PercentBlack (log)	983	2.218	0.908	-0.337	1.595	2.947	3.998
MedianInc (log)	983	3.525	0.291	2.884	3.298	3.73	4.5

Table S2: Summary Statistics: NYC

Variable	N	Mean	Std. Dev.	Min	Pctl. 25	Pctl. 75	Max
Essential	177	34.632	9.252	14.455	28.807	41.479	54.4
BAplus	177	39.855	21.89	9.091	24.532	49.004	93.188
PercentOlder	177	13.82	4.955	0	10.576	16.796	28.662
PercentYounger	177	19.966	5.51	6.26	16.63	23.294	35.282
COPD	177	6.055	1.314	2.1	5.2	6.9	9.6
Obese	177	30.206	4.818	20.7	26	34.1	39.6
OnlyEngAtHome	177	54.525	19.148	13.694	39.843	71.553	97.578
PercentWhite (log)	177	3.578	0.862	0.764	3.15	4.236	4.566
PercentHispanic (log)	177	2.988	0.765	-0.018	2.422	3.624	4.307
PercentBlack (log)	177	2.269	1.412	-1.127	1.132	3.494	4.534
MedianInc (log)	177	4.241	0.462	3.132	3.959	4.556	5.519

C The key periods

C.1 Spring - Summer 2020 – Outbreak and Confinement (begins 12 May 2020):

This is the initial period of the outbreak. London limited testing to symptomatic (and generally severe) cases, sometimes post-mortem, and in this period it was estimated that between 2 and 5 times as many cases were occurring (Pouwels et al., 2021; Silverman, Hupert, & Washburne, 2020). NYC limited its reporting to its five boroughs until May 18, at which point ZIP code level data were released, and MSOA level data were released in London. Based on this, we restrict the primary comparative analysis to begin mid-May 2020 (see also data section). By the time that we have reliable data, there has been widespread diffusion. No one knows precisely how the virus spreads, and Personal Protective Equipment (PPE) is not fully available in either city, even for healthcare workers. Inaccurate information is propagated by both governments. E.g., in the UK, the government claimed that masking does not protect against the disease; this could have been propagated to preserve the available supply for the healthcare workforce, as was done in France (VOX, 2020). In the US, confusion about potential treatments, such as hydroxychloroquine, perhaps

led to underplaying the lethal nature of the disease (AJMC Staff, 2021, discusses the early measures taken and proposed). Thus information and misinformation flooded social media outlets from the outset, and conflicting policies between federal and local governments emerged (notably on mask wearing).

Following an influential report (N. M. Ferguson et al., 2000), calls to "flatten the curve" (Mahase, 2020) were accompanied by policies such as: school closures, remote work, limited commerce, bans on indoor dining, and social distancing. Mask wearing in public settings became law, earlier in NYC (April) than in London (June). Incidence rates eventually plummeted, and while the aforementioned policies can be expected to have had significant influence (Berger et al., 2021; Brooks et al., 2020), rising temperatures likely also played a key role. The low incidence rates withstood the social upheaval in both countries, following the police killing of George Floyd in the US. The resulting protests were outdoors in higher temperatures, masks were commonly worn, and the modal age of protesters was fairly low, so the risk of serious illness remained low. By the summer, there was sufficient information available to assess one's personal risk, if only approximately.

C.2 Fall 2020 – Restarting of economies (begins 08 Sept 2020):

With incidence of more restrictive measures, such as "lockdowns," now solidly in the past, with testing measures in place and with reduced caseloads, both cities (and much of the world) sought to restart their economies in the Fall of 2020. Indoor dining began first in London, during the month of August, and the government used discount incentives to bring people back to in-person experiences. In NYC at that time, the primary approach was to construct outdoor dining facilities on sidewalks and streets. This meant that individuals had more dining options and social distancing was feasible indoors. Houses of worship were allowed to operate in person, but at limited capacity. Variations on social distancing and limiting crowds allowed cultural venues, gyms and hairdressers to open before this period began (July 4th). This "reopening" is the context for this key period (W. Ferguson et al., 2022).

Schooling began in a hybrid mode, shifting to remote for most of the school year in both cities. School didn't close in London during this period, but universities and colleges went remote from November onward. NYC schools went remote on November 18th, while nearly all college instruction was remote. Some religious holidays were associated with hot spot outbreaks in NYC (e.g., Williamsburg and Borough Park, Brooklyn; see Fitzsimmons E. & Petri A., 2020; Stack L., 2021). No comparable pattern was found in London until the Summer 2021 football matches.

C.3 Late Fall - Early Winter 2020 (second wave and lockdown) (begins 24 Nov 2020):

In this period, most businesses allowed some level of activity (e.g., restricting only the number of patrons). Unfortunately, a new outbreak (wave) emerges in conjunction with politics and policies. Contrary to the prior patterning, NYC's wave began in the prior period, with election politics, religious practice, COVID fatigue, etc., playing some role. The wave prompted a series of school closures in NYC. Just prior to this period, on Saturday October 31th, 2020, the British Prime Minister announced that a new lockdown would come into force the following Thursday (November 5th) at midnight. The lockdown should act as a fix to 'save Christmas', so would last 4 weeks only, ending shortly before Christmas, leaving enough time for families to engage in their Christmas shopping. The plan was to minimize trades' losses for retail businesses and the hospitality industry. This was rapidly followed by a spike in cases increasingly dominated by the Alpha variant (Nicholson

et al., 2022).

The time lag of 5 days between the announcement and the enforced lockdown appears to have contributed further to the spread of the virus, already widely in circulation at the time of the announcement (Hunter, Brainard, & Grant, 2021). There are reports of frantic socialization in anticipation of the new lockdown during those five days. At the time of the announcement, regions in the North East, North West and Midlands (which include many university towns) faced infection rates of 300 per 100,000 population (in London, it was 120). These students then headed home, often to London.

By the end December, the spread of the Delta (now denominated as new variant of concern, VOC) was assessed and shown to have resisted and expanded amongst younger age group, Chand et al. (2020). Hence, the lockdown while having a significant impact in reducing the spread of earlier strains (Alpha) had little effect of the spread of the Delta, possibly a result of the combined effect of transmission rate up to 4x higher, different age groups susceptibility, and the public health focus being on the North while it was transmitting in London and the South East (N. Davies et al., 2021; N. G. Davies et al., 2021).

C.4 Late Winter/early Spring 2021 (begins 23 Feb 2021):

In this period, there is a major outbreak in both communities. There are steeper gradients in London, for both paths up and down the peak, which was reached in London at the end of January. Schools shut 9 weeks (Jan 4th-Mar 8th) while nurseries stayed open. At the peak, London is a near full lockdown, which is partially lifted from early March. Covid passes (or negative test results) are no longer required in public spaces. New York City does not enter a full lockdown, but screening for fevers at entry points and mask wearing were enforced in public places. Both cities engage in an aggressive vaccination campaign, initially prioritizing older and more vulnerable individuals.

C.5 Late Spring/Early Summer 2021 (begins 11 May 2021):

In London, this is a period a rapid vaccination, with a progressive roll out to younger age groups. The vaccination is operated centrally with very strict respect of age ranges. A flight ban is introduced from India, from April 23rd, with British travelling from there allowed in the country but required to isolate in supervised 'hotels' for 10 days, which cost GBP 1750 per traveler. This rule applies to all countries on their 'red list'. The rule was to prevent the spread of the Delta variant (B.1.617) first detected on April 21st but this did not prevent a surge of cases (200k cases daily in the UK beginning April 15), and by Mid-May Delta is the dominant variant in the UK (McCrone et al., 2022). In NYC, the Delta variant is identified as a 'concern' in June, but it does not dominate the infections until after this period. Moreover, health officials drew upon data emanating from London, and variation in vaccination and masking practice across its boroughs to suggest policy (Goldstein J., 2021).

C.6 Mid-Summer - Fall 2021 (begins 13 July 2021):

The return to school in the UK has the potential to introduce a new wave in unvaccinated youth. In fact, many health officials at the time expected an increase in cases with the return as well as cooling temperatures. At this time, the Delta variant is firmly in place in NYC, and there is a clear 'bump' in cases for 4-12 and 13-18 year olds in NYC. Our examination of age-specific, publicly-available time-series from both cities at this time revealed differences by age that suggested the movement

from children to their (younger) parents. As well, younger neighborhoods of Manhattan, with college-age adults, had somewhat higher incidence rates.

C.7 Winter 2021

The Omicron variant spreads in both cities. By late December, London implements a form of partial confinement and NYC has increased mask-wearing requirements. The spread appears to be community-wide, but it moves in identifiable "waves," beginning with young adults and children, who are less fully vaccinated, and perhaps essential workers, and then on to those in close proximity to them. Our same examination of age-specific, publicly-available time-series suggested a wavelike movement through adjacent communities as well. Vaccination campaigns appear to have reduced death and to some extent hospitalization rates, as compared to prior waves, but this is a 'breakthrough' variant, despite a round of booster shots in both cities.

In the early phases of this wave, NYC residents lacked sufficient at-home test kits, and as such cases went undetected. As the wave receded, at-home test became more available (both privately and free through the government), and self-reporting of test results likely diminished, at least in NYC, where healthcare is administered through a combination of private and public care, with substantially less coordination as in the UK.

We end our study at this point in part because the data upon which we rely has become increasingly less reliable.

References

AJMC Staff. (2021). *A Timeline of COVID-19 Developments in 2020.* Retrieved from https://www.ajmc.com/view/a-timeline-of-covid19-developments-in-2020

Berger, L., Berger, N., Bosetti, V., Gilboa, I., Hansen, L. P., Jarvis, C., Marinacci, M., & Smith, R. D. (2021, 1). Rational policymaking during a pandemic. *Proceedings of the National Academy of Sciences (PNAS)*, *118*.

Brooks, S. K., Smith, L. E., Webster, R. K., Weston, D., Woodland, L., Hall, I., & Rubin, G. J. (2020, 4). The impact of unplanned school closure on children's social contact: Rapid evidence review. *Eurosurveillance*, *25*, 1-10.

Chand, M., Hopkins, S., Dabrera, G., Barclay, W., Ferguson, N., Volz, E., Loman, N., Rambaut, A., & Barrett, J. (2020, 12). Investigation of novel SARS-COV-2 variant of concern 2020/12/01. *Public Health England, Technical Briefing 2*.

Chen, Y.-H., Glymour, M., Riley, A., Balmes, J., Duchowny, K., Harrison, R., Matthay, E., & Bibbins-Domingo, K. (2021, 6). Excess mortality associated with the covid-19 pandemic among californians 18-65 years of age, by occupational sector and occupation: March through november 2020. *PLoSONE*, *16*. doi: 10.1371/journal.pone.0252454

Davies, N., Jarvis, C., Edmunds, W., Jewell, N., Diaz-Ordaz, K., & Keogh, R. (2021). Increased mortality in community-tested cases of SARS-CoV-2 lineage B. 1.1. 7. *Nature*, *593*(7858), 270–274.

Davies, N. G., Abbott, S., Barnard, R. C., Jarvis, C. I., Kucharski, A. J., Munday, J. D., Pearson, C. A. B., Russell, T. W., Tully, D. C., Washburne, A. D., Wenseleers, T., Gimma, A., Waites, W., Wong, K. L. M., van Zandvoort, K., Silverman, J. D., Diaz-Ordaz, K., Keogh, R., Eggo, R. M., Funk, S., Jit, M., Atkins, K. E., & Edmunds, W. J. (2021, 4). Estimated transmissibility and impact of SARS-CoV-2 lineage B.1.1.7 in England. *Science*, *372*(6538).

Ferguson, N. M., Laydon, D., Nedjati-Gilani, G., Imai, N., Ainslie, K., Baguelin, M., Bhatia, S., Boonyasiri, A., Cucunubá, Z., Cuomo-Dannenburg, G., Dighe, A., Dorigatti, I., Fu, H., Gaythorpe, K., Green, W., Hamlet, A., Hinsley, W., Okell, L. C., Van Elsland, S., Thompson, H., Verity, R., Volz, E., Wang, H., Wang, Y., Gt Walker, P., Walters, C., Winskill, P., Whittaker, C., Donnelly, C. A., Riley, S., & Ghani, A. C. (2000). *Report 9: Impact of non-pharmaceutical interventions (NPIs) to reduce COVID-19 mortality and healthcare demand* (Tech. Rep.). London: Imperial College.

Ferguson, W., Furticella, J., Hinderaker, A., Howard, H., Newman, A., & Rogers, K. (2022, 3). Two Years of the Pandemic in New York, Step by Awful Step. *New York Times*. Retrieved from https://www.nytimes.com/interactive/2022/nyregion/nyc-covid-timeline.html

Fitzsimmons E., & Petri A. (2020). *How Staten Island Became a Coronvirus Hot Spot.* Retrieved from https://www.nytimes.com/2020/11/11/nyregion/staten-island-second-wave.html

Goldstein J. (2021). *As Delta Variant Spreads, Coronavirus Cases in NYC Rise -.* Retrieved from https://www.nytimes.com/2021/07/14/nyregion/delta-variant-coronavirus.html

Huang, J., Brink, H. M. V. D., & Groot, W. (2011). College education and social trust: An evidence-based study on the causal mechanisms. *Social Indicator Research*, *104*, 287-310.

Hunter, P. R., Brainard, J., & Grant, A. (2021). The Impact of the November 2020 English National Lockdown on COVID-19 case counts. *medRxiv*.

Mahase, E. (2020). Covid-19: Uk starts social distancing after new model points to 260 000 potential deaths. *BMJ*, *368*.

McCrone, J. T., Hill, V., Bajaj, S., Pena, R. E., Lambert, B. C., Inward, R., Bhatt, S., Volz, E., Ruis, C., Dellicour, S., Baele, G., Zarebski, A. E., Sadilek, A., Wu, N., Schneider, A., Ji, X., Raghwani, J., Jackson, B., Colquhoun, R., O'Toole, , Peacock, T. P., Twohig, K., Thelwall,

S., Dabrera, G., Myers, R., Faria, N. R., Huber, C., Bogoch, I. I., Khan, K., du Plessis, L., Barrett, J. C., Aanensen, D. M., Barclay, W. S., Chand, M., Connor, T., Loman, N. J., Suchard, M. A., Pybus, O. G., Rambaut, A., & Kraemer, M. U. G. (2022, 8). Context-specific emergence and growth of the SARS-CoV-2 Delta variant. *Nature 2022*, 1–3.

Nafilyan, V., Pawelek, P., Ayoubkhani, D., Rhodes, S., Pembrey, L., Matz, M., Coleman, M., Allemani, C., Windsor-Shellard, B., van Tongeren, M., & Pearce, N. (2022). Occupation and covid-19 mortality in england: a national linked data study of 14.3 million adults. *Occupational and Environmental Medicine*, *79*, 433-441.

Nicholson, G., Lehmann, B., Padellini, T., Pouwels, K. B., Jersakova, R., Lomax, J., King, R. E., Mallon, A.-M., Diggle, P. J., Richardson, S., Blangiardo, M., & Holmes, C. (2022). Improving local prevalence estimates of SARS-CoV-2 infections using a causal debiasing framework. *Nature Microbiology*, *7*, 97–107.

ONS. (2020). *Which occupations have the highest potential exposure to the coronavirus (COVID-19)? - Office for National Statistics.* Retrieved from `https://www.ons.gov.uk/employmentandlabourmarket/peopleinwork/employmentandemployeetypes/articles/whichoccupationshavethehighestpotentialexposuretothecoronaviruscovid19/2020-05-11`

Pouwels, K. B., House, T., Pritchard, E., Robotham, J. V., Birrell, P. J., Gelman, A., Vihta, K. D., Bowers, N., Boreham, I., Thomas, H., Lewis, J., Bell, I., Bell, J. I., Newton, J. N., Farrar, J., Diamond, I., Benton, P., Walker, A. S., Pouwels, K. B., Walker, A. S., Crook, D., Matthews, P. C., Peto, T., Stoesser, N., Howarth, A., Doherty, G., Kavanagh, J., Chau, K. K., Hatch, S. B., Ebner, D., Martins Ferreira, L., Christott, T., Marsden, B. D., Dejnirattisai, W., Mongkolsapaya, J., Hoosdally, S., Cornall, R., Stuart, D. I., Screaton, G., Eyre, D., Bell, J., Cox, S., Paddon, K., James, T., Newton, J. N., Robotham, J. V., Birrell, P., Jordan, H., Sheppard, T., Athey, G., Moody, D., Curry, L., Brereton, P., Hay, J., Vansteenhouse, H., Lambert, A., Rourke, E., Hawkes, S., Henry, S., Scruton, J., Stokes, P., Thomas, T., Allen, J., Black, R., Bovill, H., Braunholtz, D., Brown, D., Collyer, S., Crees, M., Daglish, C., Davies, B., Donnarumma, H., Douglas-Mann, J., Felton, A., Finselbach, H., Fordham, E., Ipser, A., Jenkins, J., Jones, J., Kent, K., Kerai, G., Lloyd, L., Masding, V., Osborn, E., Patel, A., Pereira, E., Pett, T., Randall, M., Reeve, D., Shah, P., Snook, R., Studley, R., Sutherland, E., Swinn, E., Tudor, A., Weston, J., Leib, S., Tierney, J., Farkas, G., Cobb, R., Van Galen, F., Compton, L., Irving, J., Clarke, J., Mullis, R., Ireland, L., Airimitoaie, D., Nash, C., Cox, D., Fisher, S., Moore, Z., McLean, J., & Kerby, M. (2021, 1). Community prevalence of SARS-CoV-2 in England from April to November, 2020: results from the ONS Coronavirus Infection Survey. *The Lancet Public Health*, *6*(1), e30-e38.

Silverman, J. D., Hupert, N., & Washburne, A. D. (2020). Using influenza surveillance networks to estimate state-specific prevalence of sars-cov-2 in the united states. *Science Translational Medicine*, *12*(554), eabc1126.

Smith, G. D., Hart, C., Hole, D., Mackinnon, P., Gillis, C., Watt, G., Blane, D., & Hawthorne, V. (1998). Education and occupational social class: which is the more important indicator of mortality risk? *J Epidemiol Community Health*, *52*, 153-160.

Stack L. (2021). *Backlash Grows in Orthodox Jewish Areas Over Virus Crackdown by Cuomo.* Retrieved from `https://www.nytimes.com/2020/10/07/nyregion/orthodox-jews-nyc-coronavirus.html`

Tessum, C. W., Paolella, D. A., Chambliss, S. E., Apte, J. S., Hill, J. D., & Marshall, J. D. (2021, 4). Pm2.5 polluters disproportionately and systemically affect people of color in the united states. *Science Advances*, *7*, 4491-4519.

The Health Foundation. (2020). *Black and minority ethnic workers make up a disproportionately*

large share of key worker sectors in London.

VOX. (2020). *How France's President Emmanuel Macron bungled his coronavirus response - Vox.* Retrieved from https://www.vox.com/2020/4/14/21218927/coronavirus-covid-france-macron-response
